# Hepatocellular Carcinoma Incidences and Risk Factors in Hepatitis C Patients: Interferon versus Direct-Acting Agents

**DOI:** 10.3390/v16091485

**Published:** 2024-09-18

**Authors:** Yu-Ting Kao, Yen-Chun Liu, Ya-Ting Cheng, Yu-Wen Wen, Yi-Chung Hsieh, Cheng-Er Hsu, Chung-Wei Su, Jennifer Chia-Hung Tai, Yi-Cheng Chen, Wen-Juei Jeng, Chun-Yen Lin, Rong-Nan Chien, Dar-In Tai, I-Shyan Sheen

**Affiliations:** 1 Department of Gastroenterology and Hepatology, Chang Gung Memorial Hospital, Linkou Branch, Taoyuan 333, Taiwan; sagittarius20025@gmail.com (Y.-T.K.); yenchun923@gmail.com (Y.-C.L.); missbear123@yahoo.com.tw (Y.-T.C.); cutebuw@yahoo.com.tw (Y.-C.H.); sjuujs2001@gmail.com (C.-E.H.); willy88926@gmail.com (C.-W.S.); heykid@livemail.tw (J.C.-H.T.); yichengliver@gmail.com (Y.-C.C.); chunyenlin@gmail.com (C.-Y.L.); ronald@cgmh.org.tw (R.-N.C.); taidi0027@gmail.com (D.-I.T.); happy95kevin@gmail.com (I.-S.S.); 2 School of Medicine, College of Medicine, Chang Gung University, Taoyuan 333, Taiwan; 3 Department of Biomedical Sciences, College of Medicine, Chang Gung University, Taoyuan 333, Taiwan; ywwen@mail.cgu.edu.tw; 4 Department of Thoracic Surgery, Chang Gung Memorial Hospital, Taoyuan 333, Taiwan

**Keywords:** hepatocellular carcinoma, Hepatitis C virus, sustained viral response, direct-acting antivirals, interferon-based therapy

## Abstract

Background: Hepatocellular carcinoma (HCC) remains a significant concern for patients with chronic hepatitis C (HCV), even after achieving a sustained virological response (SVR) with direct-acting antivirals (DAAs) or interferon (IFN)-based therapies. This study compared the risk of HCC in patients with HCV who achieved SVR through the DAA versus IFN regimens. Methods: A retrospective analysis was conducted on 4806 HCV patients, without coinfection nor prior HCC history, treated at the Chang Gung Memorial Hospital, Taiwan (DAA: 2825, IFN: 1981). Kaplan–Meier and Cox regression analyses with propensity score matching (PSM) were used to adjust for baseline differences. Results: DAA-treated patients exhibited a higher incidence of HCC than IFN-treated patients before and after PSM (after PSM: annual: 1% vs. 0.5%; 6-year: 6% vs. 3%, *p* = 0.01). Both DAA and IFN patients had a decreased HCC incidence during follow-up (>3 vs. <3 years from the end of treatment: DAA: 1.43% vs. 1.00% per year; IFN: 0.47% vs. 0.36% per year, both *p* < 0.05). HCC incidence was higher in the first three years post-SVR in DAA-treated ACLD patients and then decreased (3.26% vs. 1.39% per year, *p* < 0.01). In contrast, HCC incidence remained constant in the non-ACLD and IFN-treated groups. Multivariate Cox regression identified age ≥ 60, male sex, BMI, AFP ≥ 6 ng/mL, FIB-4, and ACLD status as independent risk factors for HCC, but antiviral regimens were not an independent factor for HCC. Conclusion: DAA treatment significantly affects HCC risk primarily within three years post-treatment, especially in younger HCV patients with ACLD. HCC incidence was reduced after three years in ACLD patients treated by DAA, but continued surveillance was still necessary. However, patients under 60 without advanced liver disease may require less intensive follow-up.

## 1. Introduction

Hepatocellular carcinoma (HCC) is a significant global health burden closely linked to chronic hepatitis C virus (HCV) infection [1]. Historically, interferon-based therapies have effectively reduced HCC risk but are constrained by considerable side effects, reduced sustained virological response (SVR) rates in genotype 1 or cirrhotic patients, prolonged treatment durations, and common side effects of flu-like symptoms, nausea, fatigue, and elevated transaminases. In recent years, direct-acting antivirals (DAAs) have emerged as the primary treatment modality for hepatitis C, achieving an SVR rate exceeding 90% with fewer side effects, shorter treatment courses, and overcoming the aforementioned treatment limitations [2,3]. DAAs also was proven to be more effective than interferon-based therapy in reducing HCV-related extrahepatic manifestations [4,5,6].

Although achieving the WHO goal of viral elimination by 2030 may be feasible for HCV patients, the remnant risk of HCC in SVR HCV patients persists. Despite the advances brought about by DAAs, it remains uncertain how these treatments compare to interferon in terms of HCC risk, particularly given that DAAs lack the immunomodulatory effects of interferon, which might influence HCC incidence in both the short- and long-term [7,8]. Most existing studies, including systemic reviews and meta-analyses, do not offer comparative data within the same population or center, leaving a gap in understanding and clarifying the confounding impact of baseline demographic differences [9,10]. Few studies have provided comparative data between the two regimens, but their follow-up duration for the DAA arm is often too short (less than 2–3 years) to allow for a comprehensive comparison with IFN-based regimens [11,12,13,14,15,16].

This study aimed to compare the incidence of HCC in HCV patients who achieved SVR through different treatments (DAAs versus interferon), especially in different subgroup populations, including their pretherapy advanced chronic liver disease (ACLD) status, different age groups, early onset (<3 years) and late onset (≥3 years), and whether HCC risk factors differ due to their antiviral regimens.

## 2. Materials and Methods

### 2.1. Patient Recruitment

We included chronic HCV infection treated with DAAs between 2015 and 2020, and 2471 patients were treated with an interferon-based regimen (interferon or pegylated interferon with or without ribavirin) between 1988 and 2017 at Chang Gung Memorial Hospital, Linkou branch, Taiwan. Patients who did not complete treatment or achieve SVR, were lost to follow-up after the assessment of SVR, had HIV or HBV co-infection, were diagnosed with HCC before DAA therapy or before SVR, or whose pretherapy FIB-4 was not available were excluded. Patients who received interferon plus DAAs were not enrolled in the current analysis. A total of 4806 patients were enrolled in the analysis (IFN, 1981; DAA, 2825) (Supplementary Figure S1). 

### 2.2. Clinical Parameters and ACLD Diagnosis

Pretreatment characteristics, including age, sex, body mass index (BMI), DAA regimens, HCV genotype and viral load, alanine aminotransferase (ALT), total bilirubin, albumin, platelet count, alpha-fetoprotein (AFP), model for end-stage liver disease (MELD) score, diabetes mellitus, and fatty liver were assessed. The FIB-4 index was obtained before starting antiviral therapy and calculated using the following formula:

FIB-4 = age [years] × AST [IU/L]/platelet count [platelets × 10^9^/L] × (ALT^1/2^[IU/L]).

“Advanced chronic liver disease (ACLD)” was defined as a pretherapy FIB-4 index ≥ 3.25 or consecutive ultrasonography evidence of cirrhosis. Hepatic decompensation was defined as a severe clinical syndrome with jaundice (total bilirubin ≥ 2 mg/dL) and INR ≥ 1.5 and/or occurrence of ascites/encephalopathy/variceal bleeding.

### 2.3. Diagnosis of HCC and HCC Surveillance

As Taiwan’s health insurance covers HCC surveillance for all HCV patients regardless of ACLD status, with even more frequent intervals of 3–4 months for cirrhotic patients, all subjects in this cohort underwent abdominal ultrasonography performed by experienced experts before initiating antiviral therapy. After the end of antiviral therapies, all HCV patients underwent regular HCC surveillance with ultrasonography exam and serum alpha-fetoprotein measurements every 3–6 months for ACLD patients and every 6–12 months for non-ACLD patients. De novo HCC was diagnosed by contrast dynamic imaging, including triphasic computed tomography (CT), magnetic resonance imaging (MRI), and/or histological proof. The HCC surveillance strategy/policy and de novo HCC diagnosis criteria remained consistent from the interferon-based regimen era to the DAA era in the current study. Early-onset HCC was defined as HCC occurrence within three years from the end of therapy (EOT); late-onset HCC was defined as HCC occurrence three years after EOT.

### 2.4. Statistical Analysis

Demographic features were compared between the different DAA and IFN groups as well as those with and without de novo HCC development. Continuous variables were summarized as means ± SD or medians (interquartile range) and analyzed by independent Student’s *t*-test or Mann–Whitney U test according to their normality distribution. Categorical variables were summarized using frequencies and analyzed using the chi-square or Fisher’s exact test. The Youden index was used to determine the optimal cut-off of continuous variables, such as AFP, with the greatest sensitivity and specificity associated with HCC occurrence. The follow-up duration was calculated from the end of treatment to de novo HCC occurrence or the last follow-up. Those who developed de novo HCC within three months after treatment cessation were censored, as the impact of antiviral therapy on HCC development may not have occurred yet, and these cases of carcinogenesis might have resulted from baseline liver conditions. Cumulative incidences of HCC were analyzed using the Kaplan–Meier method and compared between groups using the log-rank test. To compare the cumulative incidence of HCC before and after three years of SVR, the Fine–Grey regression model was applied to compare the incidence of <3 years versus ≥3 years, which was significantly different; the cut-off of three years was used because the average follow-up duration in the DAA group was approximately three years. Multivariate Cox regression analysis was used to identify independent risk factors for HCC. Cox hazard regression was also applied to predictors of early HCC (within three years after EOT) and late HCC (3–6 years from EOT) by the corresponding time scale (Supplementary Figure S2). The risks of early and late HCC in patients treated with DAA and IFN-treated patients were compared using the Cox hazard regression. Propensity score matching (PSM) at a 1:1 ratio was applied to adjust for the characteristic differences between DAA- and IFN-treated patients, including age, sex, HCV genotypes, pretherapy FIB-4 levels, ACLD status, and HCV RNA level, to reduce the potential bias in the treatment comparison estimate due to possible observed confounders. Variables with *p* < 0.05 in the univariate regression analysis were included in the multivariate regression model. Statistical analyses were conducted using SAS software (version 9.4; SAS Institute, Inc., Cary, NC, USA) and R.

## 3. Results

### 3.1. Clinical Characteristics of DAA- and IFN-Treated Patients

Between 2015 and 2020, 3763 HCV patients were treated with DAAs, and 2471 patients were treated with an interferon-based regimen between 1988 and 2017. Among enrolled 4806 HCV patients who completed antiviral treatment with SVR, 2825 (59%) were treated with DAAs, and 1981 (41%) were treated with IFN-based regimens. The mean age was 58 (±13) years, 48% were male, 56% were infected with genotype 1, 36% had ACLD, and the median follow-up duration was 54 months (IQR: 36–137) month (Table 1). Compared to IFN-treated patients, the DAA group was older (62 vs. 52 years, *p* < 0.001), had a lower proportion of males (43% vs. 57%, *p* < 0.001), and had higher proportions of genotype 1 (60% vs. 49%, *p* < 0.001), type 2 diabetes mellitus (18% vs. 9%, *p* < 0.001), ACLD (38% vs. 33%, *p* = 0.001), and pretherapy hepatic decompensation (0.5% vs. 0, *p* < 0.001). They also had higher pretherapy HCV RNA levels, lower levels of ALT, AST, AFP, albumin, and MELD scores, and shorter follow-up durations (median: 42 vs. 152 months) (Table 1). After propensity score matching (PSM) adjusted for baseline characteristics, age, sex, HCV genotype, HCV RNA level, and liver fibrotic status, there were 1317 patients in each of the DAA and IFN groups, with comparable age, proportions of males, HCV genotype 1, ACLD status, and levels of HCV RNA and FIB-4 (Supplementary Table S1).

### 3.2. HCC Incidence Stratified by ACLD Status and Age

The 6-year cumulative incidence of HCC was significantly higher in patients aged ≥ 60 years or older compared to those aged < 60 years, regardless of ACLD status (ACLD: age ≥ 60 vs. < 60 years: 12% vs. 8%, log-rank *p* = 0.012; non-ACLD: age ≥ 60 vs. < 60 years: 3% vs. 0.4%, log-rank *p* < 0.001) (Supplementary Figure S3). Patients with ACLD aged ≥ 60 years had the highest annual incidence of HCC, followed by patients with ACLD aged < 60 years. Non-ACLD patients had a lower HCC risk, with the lowest risk observed in non-ACLD patients under 60 years of age, a trend validated in both the DAA and IFN groups (Table 2).

### 3.3. HCC Incidences in DAA and IFN-Treated Patients

**Figure 1:**
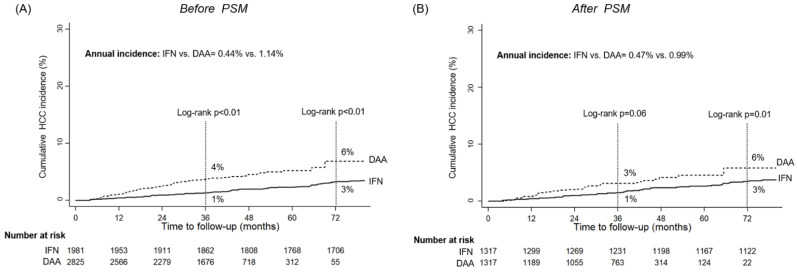
The cumulative HCC incidences in interferon (IFN)- and direct-acting antivirals (DAA)-treated patients. (**A**) before propensity score matching (PSM) (**B**) after PSM.

In the DAA and IFN-treated groups, the median follow-up durations were 42 (30–51) and 152 (111–191) months, respectively, and after achieving SVR, 106 and 102 patients developed HCC. Before PSM, the annual 3- and 6-year cumulative incidences of HCC were significantly higher in the DAA group than in the IFN-based regimen (annual: 1.14% vs. 0.44%, 3-year: 4% vs. 1%, 6-year: 6% vs. 3%, all log-rank *p* < 0.01) (Figure 1A). After PSM, the 3-year cumulative incidence of HCC was higher in the DAA groups than in the IFN groups (3% vs. 1%, log-rank *p* = 0.06). However, the 6-year cumulative incidence of HCC was significantly greater in the DAA group (6% vs. 3%, *p* = 0.01) (Figure 1B).

The impact of treatment regimens on HCC incidence primarily existed during the early follow-up period (within three years from EOT), with the DAA group showing a three-fold higher incidence than IFN-treated patients (1.43% vs. 0.47%). However, these differences diminished three years after EOT (DAA vs. IFN: 1.00% vs. 0.36% per year) (Supplementary Figure S4).

### 3.4. Impact of Antiviral Regimens on HCC Incidence Varies by ACLD and Age Status

**Figure 2:**
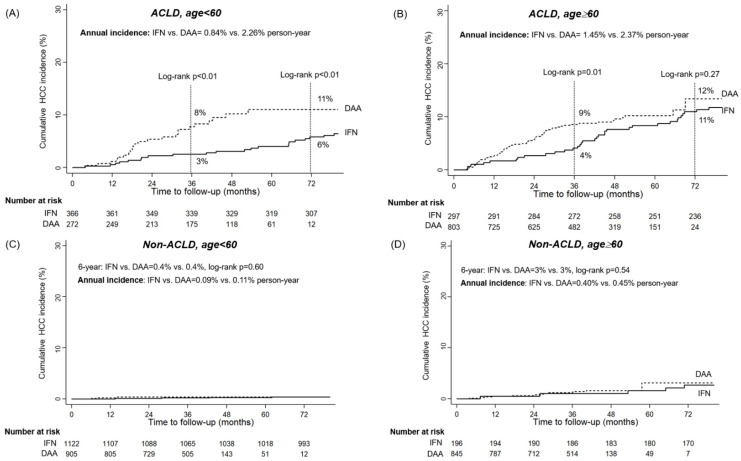
The comparisons of the cumulative HCC incidences in interferon (IFN)- and direct-acting antivirals (DAA)-treated patients stratified by liver fibrotic stages and ages before PSM. (**A**) in advanced chronic liver disease (ACLD) patients with age < 60 years (**B**) in ACLD patients with age ≥ 60 years (**C**) in non-ACLD patients with age < 60 years (**D**) in non-ACLD patients with age ≥ 60 years.

**Figure 3:**
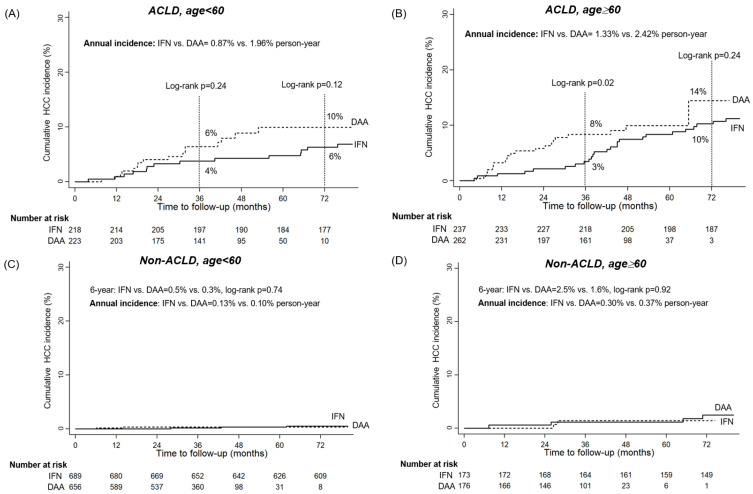
The comparisons of the cumulative HCC incidences in interferon (IFN)- and direct-acting antivirals (DAA)-treated patients stratified by liver fibrotic stages and ages after PSM. (**A**) in advanced chronic liver disease (ACLD) patients with age < 60 years (**B**) in ACLD patients with age ≥ 60 years (**C**) in non-ACLD patients with age < 60 years (D) in non-ACLD patients with age ≥ 60 years.

Younger DAA-treated patients with ACLD had a significantly higher incidence compared to their IFN-treated counterparts, with a 6-year cumulative incidence of 11% versus 6% and an annual incidence of 2.26% versus 0.84% (log-rank *p* < 0.001) (Figure 2A). These differences were more pronounced after three years of follow-up in the PSM-matched cohort (Figure 3A). In older ACLD patients (>60 years), the treatment effect was more evident in the short term (3-year: 4% versus 9%, before PSM, *p* = 0.01; 3% versus 8% after PSM, *p* = 0.02) than in the long term (6-year: 11% versus 12% before PSM, *p* = 0.27; 10% versus 14% after PSM, *p* = 0.24) (Figure 2B and Figure 3B). The treatment regimen had a minimal impact on HCC incidence in non-ACLD patients before and after PSM (Figure 2C,D and Figure 3C,D). Younger CHC patients without ACLD who achieved SVR, regardless of the antiviral regimen, had a very low annual HCC incidence (<0.2%), suggesting that less frequent HCC surveillance programs might be sufficient for this group (Figure 2C and Figure 3C).

### 3.5. Predictors of HCC Occurrence in DAA- and IFN-Treated Patients

Among the 4806 HCV patients treated with either DAA or IFN-based therapy, multivariate Cox regression identified several independent factors associated with HCC occurrence: age ≥ 60 years [adjusted hazard ratio (aHR): 1.888 (95% CI: 1.263–3.085), *p* = 0.001], male sex [aHR: 2.481 (1.770–3.476), *p* < 0.001], higher BMI [aHR: 1.056 (1.012–1.102), *p* = 0.013], pretherapy AFP level ≥ 6 ng/mL [aHR: 3.034 (2.005–4.591), *p* < 0.001], higher pretherapy FIB-4 level [aHR: 1.056 (1.020–1.093), *p* = 0.002], and ACLD status [aHR: 3.297 (2.012–5.403), *p* < 0.001]. Conversely, higher albumin levels were associated with a lower risk of HCC (aHR: 0.631 [0.429–0.927], *p* = 0.019) (Table 3). Univariate analysis showed a higher crude HR for HCC in the DAA group than in the IFN group [crude HR: 2.264 (1.607–3.191) *p* < 0.001), and the antiviral regimen itself was not an independent factor after adjusting for other variables [aHR: 1.434 (0.875–2.351), *p* = 0.152]. These findings were consistent in the PSM-matched cohort, with the exception that genotype 2 CHC patients had a lower risk of HCC than genotype 1 (aHR: 0.489 [0.283–0.846], *p* = 0.011) (Supplementary Table S2).

When analyzed by antiviral regimen, the common risk factors for HCC in both groups were higher AFP and ACLD status. However, in the IFN group, age ≥ 60 years was an additional independent risk factor, whereas, in the DAA group, male sex and higher FIB-4 levels were also significant predictors of HCC (Table 4).

### 3.6. Comparison of Predictors and Incidence between Early-Onset and Late-Onset HCC

Multivariate Cox regression identified older age (≥60 years), AFP ≥ 6 ng/mL, and ACLD status as common factors associated with both early- (<3 years) and late-onset (>3 years) HCC (Supplementary Tables S3 and S4). Notably, DAA treatment was a significant predictor of early onset HCC (aHR: 2.373 [1.147–4.911], *p* = 0.020) but not late HCC.

**Figure 4:**
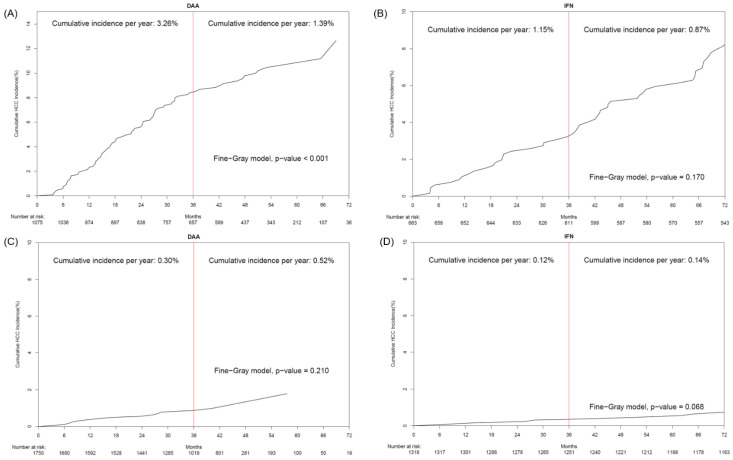
The comparisons of the HCC risks within three years (early onset) and after three years (late onset). (**A**) ACLD patients treated by DAA (**B**) ACLD patients treated by IFN (**C**) non-ACLD patients treated by DAA (**D**) non-ACLD patients treated by IFN.

Both DAA and IFN arms showed that the HCC incidence decreased after three years from EOT (early vs. late HCC annual incidence: DAA 1.43% vs. 1.00% per year, IFN: 0.47% vs. 0.36% per year, both *p* value < 0.05 by Fine-Gray model, Supplementary Figure S4). However, among ACLD patients treated with DAA, the annual incidence of early-onset HCC was twice as high as that of late-onset HCC (early versus late HCC annual incidence: 3.26% vs. 1.39% per year, Fine-Gray *p* < 0.001, Figure 4). In contrast, early and late HCC risks were comparable among patients with and without IFN-treated ACLD (Figure 4, Table 2).

## 4. Discussion

In the era of high viral eradication rates achieved by direct-acting antivirals (DAA) in chronic hepatitis C, the risks of hepatitis-related adverse outcomes, especially hepatocellular carcinoma (HCC), diminish after the sustained virological response (SVR). Despite this, HCC remains a significant health concern in patients with HCV, even after SVR. This real-world retrospective cohort study clearly demonstrated the impact of different antiviral regimens on post-SVR de novo HCC development, proposing both short-term and long-term HCC incidence up to six years and identifying regimen-specific HCC risk factors.

HCC surveillance remains critical for HCV patients even after achieving SVR due to ongoing immunological challenges; the normal immunologic homeostasis was not restored completely, and the epigenetic scars after SVR are still left behind [17,18]. Research indicates that although DAAs effectively clear HCV, they also reduce the cytotoxic function of natural killer (NK) cells and suppress intrahepatic macrophage and MAIT cell activity without restoring normal cytokine production. These changes may impair the immune system’s ability to target and eliminate cancer cells. Additionally, DAAs do not normalize the increased frequencies of regulatory T cells (Tregs), which can further hinder the clearance of potent cancer cells even after SVR. This further hampers the clearance of cancer cells [19,20,21,22], leading to a higher risk of HCC in DAA-treated patients than in those treated with IFN. While IFN treatment is known for its antiviral and immunomodulatory effects, recent studies involving genotype 1 HCV patients treated with DAAs alone or in combination with IFN/ribavirin have shown only a temporary expansion of activated Foxp3+ CD25+ CD4+ T cells in the DAA plus IFN group [23]. This may also explain why the impact of antiviral regimens on HCC occurrence is mainly observed in the early period, with the difference becoming negligible after three years in the current study. Neither treatment fully restored the function or activation of Tregs even a year after viral elimination, suggesting that this persistent immunosuppression could influence long-term outcomes and contribute to the risk of HCC occurrence [23].

The debate over whether DAAs reduce HCC incidence or recurrence compared with IFN treatment remains unresolved. Early studies, limited by small sample sizes and shorter follow-up periods, especially in the DAA arms, suggested that DAAs might have a limited impact on reducing HCC risk in cirrhotic HCV patients and could even increase short-term HCC risk [12,24]. A large North American cohort study reported a higher HCC incidence in the DAA group (0.69%) than in the IFN group (0.18%), with annual HCC incidence rates of 2.07% for DAA-treated and 1.5% for IFN-treated cirrhotic patients. However, the shorter follow-up duration for DAA-treated patients (typically less than two years) compared to IFN-treated patients (>3 years) complicates accurate long-term risk assessment [15,16]. In our study, with longer follow-up durations, we observed a higher HCC incidence during the early follow-up period (<3 years) than in the late follow-up period (>3 years) in both DAA and IFN treatment patients (Supplementary Figure S4). This warrants the observation that HCC surveillance in SVR patients is important during the first three years. Furthermore, a higher HCC incidence was observed in DAA-treated patients than in those treated with IFN, both before and after PSM (6-year: 6% vs. 3%, log-rank *p* < 0.01), particularly in the ACLD groups. Our findings also showed that ACLD patients treated with DAAs had a higher early HCC risk than those treated with IFN (average annual incidence: 3.26% vs. 1.15%) but a similar late HCC risk between the two groups (1.39% vs. 0.87%). We demonstrated that in ACLD patients treated with DAA, the average annual incidence of early HCC significantly exceeded that of late HCC and remained above the cost-effectiveness threshold for HCC (>1%). Conversely, the incidence differences between non-ACLD and ACLD patients treated with IFN were insignificant.

In contrast to a recent study of 17,187 HCV patients with a combined 104,000 interval years of follow-up, which reported that achieving SVR with DAAs reduced HCC risk more than IFN (aHR: 0.29, 95% CI: 0.17–0.48), our study confirmed that the treatment regimen itself may not be an independent risk factor for HCC (aHR: 1.43, 95% CI: 0.88–2.35) after adjusting for pretherapy age, sex, viral factors, fibrosis status, and other variables. This finding is consistent with those of previous cohort studies and meta-analyses [9,15,16,25]. For example, a Japanese cohort of 609 HCV patients treated with either Simeprevir plus IFN or sofosbuvir with ledipasvir found a comparable HCC incidence between the two groups after propensity score matching, with a median follow-up of five years [25]. Meta-analyses and meta-regressions have shown no significant differences in HCC occurrence and recurrence between patients receiving DAA or IFN therapies [10]. The differences across studies emphasize the importance of extended follow-up, and representative studies are essential to clarify the impact of DAAs versus IFN on HCC incidence in HCV-SVR patients [9]. However, stratified by three years after the end of treatment, DAA treatment was an independent risk factor compared to IFN for early (<3 years) HCC occurrence (aHR: 2.373, *p* = 0.02, Supplementary Table S3), whereas the treatment regimen was not an independent risk factor for late (>3 years) HCC occurrence (Supplementary Table S4). The regimen impact was more prominent in younger (<60 years) patients with ACLD (annual incidence: 2.26% versus 0.84%). These findings suggest that younger patients with ACLD treated with DAA require more vigilant HCC surveillance.

For patients treated with DAA or IFN, common HCC predictors included higher fibrosis stages, older age, male sex, higher AFP levels, and lower albumin or platelet levels [14,26,27,28,29,30,31], consistent with our Cox regression model. However, when analyzing risk factors by treatment regimen, only higher AFP and ACLD levels were common risk factors for HCC across both groups. This aligns with the current HCC surveillance recommendations, which target subjects with ACLD as the primary surveillance population. Our findings in Table 4 suggest the potential for personalized risk stratification based on an antiviral regimen: older age should be considered a risk factor in IFN-treated patients, while male sex, BMI, and FIB-4 could help identify those at risk for HCC in addition to ACLD. Contrary to previous findings that genotype 3 is a risk factor for HCC [27], our PSM-matched cohort showed that patients with genotype 2 were less likely to develop HCC. Among all subgroups, HCC incidence was highest in ACLD patients younger than 60 years (annual incidence: 2.26%), especially when treated with DAA. Subjects younger than 60 years who are non-ACLD represent the only subgroups with the lowest risk and may warrant more relaxed HCC surveillance considerations.

Several limitations of these studies must be considered. First, due to the retrospective nature of this study, we could not examine the impact of the reported SNPs, such as PBPLA3, TM6SF2, et al., on HCC development in IFN- and DAA-SVR HCV patients [32]. Second, cirrhotic patients were not stratified according to the existence of decompensation or Child-Pugh score. Third, the impact of pretherapy distinct differences in the characteristics between IFN and DAA groups may not be completely attenuated by PSM, especially when DAA groups are much older than IFN groups, which poses a higher probability of HCC development. However, the distribution of propensity scores after PS matching shows a standard deviation difference of 0.17 in both arms, representing a small biological difference [33], the impact of residual confounding under propensity matching may be minimal in the current study. Fourth, due to the lack of linkage to the national cancer registry dataset and the hospital-based cohort design, ascertainment bias may exist for cancer development if diagnosed at another hospital. However, such events may occur in patients who discontinue follow-up at our site or transfer to another hospital, with their data censored at their last visit. Finally, the shorter follow-up periods for DAA-treated patients compared to those treated with IFN complicate the long-term risk assessments.

## 5. Conclusions

Our findings indicated that DAA treatment significantly influences HCC occurrence primarily within the first three years post-treatment, with the highest risk observed in younger patients (<60 years) with HCV and ACLD. These individuals required stringent HCC surveillance during this critical period. While HCC incidence declines after three years, it remains above the cost-effectiveness threshold for HCC surveillance, supporting the need for continued monitoring. In contrast, non-ACLD patients under 60 years of age showed the lowest risk, suggesting the possibility of reduced surveillance in this subgroup. The type of treatment regimen was not relevant to long-term HCC occurrence, with the achievement of SVR being more crucial than the specific regimen used. As the HCC risk was lowest in young, non-ACLD CHC patients, treating them earlier while they are in a less fibrotic state may be the most important factor/cost-benefit policy in reducing HCC occurrence.

## Figures and Tables

**Table 1:** Comparisons of baseline clinical characteristics between patients treated with DAA and IFN.

	All, N = 4806	DAA N = 2825	IFN-Based N = 1981	*p* Value
Age, years (SD)	58 (13)	62 (12)	52 (12)	<0.001
≥60, n (%)	2141 (45)	1648 (58)	493 (25)	<0.001
Male, n (%)	2330 (48)	1204 (43)	1126 (57)	<0.001
BMI, kg/m^2^ (IQR)	24 (22–27)	24 (22–27)	24 (22–26)	0.119
HCV RNA, log_10_IU/mL (IQR)	6.1 (5.4–6.6)	6.2 (5.5–6.6)	5.9 (5.2–6.5)	<0.001
Genotype 1, n (%)	2610 (56)	1674 (60)	936 (49)	<0.001
2	1893 (40)	989 (35)	904 (48)	
3	40 (1)	23 (1)	17 (1)	
others	140 (3)	109 (4)	31 (2)	
ALT, U/L (IQR)	66 (38–123)	51 (30–96)	92 (57–153)	<0.001
AST, U/L (IQR)	52 (34–87)	46 (32–76)	63 (40–101)	<0.001
Total bilirubin, mg/dL (IQR)	0.7 (0.5–0.9)	0.7 (0.5–0.9)	0.8 (0.6–1.0)	<0.001
Albumin, g/dL (IQR)	4.4 (4.1–4.6)	4.3 (4.0–4.5)	4.5 (4.3–4.7)	<0.001
Platelet, 10^3^/uL (IQR)	178 (136–221)	181 (135–227)	173 (137–213)	<0.001
AFP, ng/mL (IQR)	4 (3–8)	4 (3–7)	5 (3–9)	<0.001
HbA1c (IQR)	5.7 (5.4–6.2)	5.8 (5.4–6.4)	5.5 (5.3–5.9)	<0.001
Diabetic mellitus, n (%)	691 (14)	505 (18)	186 (9)	<0.001
Fatty liver, n (%)	2290 (48)	1313 (47)	977 (49)	0.074
FIB-4 (IQR)	2.24 (1.41–3.57)	2.34 (1.51–3.87)	2.07 (1.26–3.20)	<0.001
≥3.25, n (%)	1405 (29)	923 (33)	482 (24)	<0.001
ACLD, n (%)	1738 (36)	1075 (38)	663 (33)	0.001
MELD score (IQR)	8.6 (7.3–10.2)	8.5 (7.2–10.2)	8.8 (7.7–10.1)	<0.001
Hepatic decompensation * (%)	15 (0.3)	15 (0.5)	0	<0.001
Follow-up, months (IQR)	54 (36–137)	42 (30–51)	152 (111–191)	<0.001

The *p* value refers to the comparison between DAA versus IFN groups. * Hepatic decompensation was defined as a severe clinical syndrome with jaundice (total bilirubin ≥ 2 mg/dL) and INR ≥ 1.5 and/or occurrence of ascites/encephalopathy/variceal bleeding. Numerical variables were expressed as mean ± standard deviation or median (interquartile range) according to their normality. ACLD, advanced chronic liver disease (defined as FIB-4 ≥ 3.25 and/or ultrasound signs of cirrhosis); AFP, alpha-fetoprotein; ALT, alanine aminotransferase; BMI, body mass index; DAA, direct-acting antivirals; HbA1c, glycohemoglobin; HCV, hepatitis C virus; IFN, interferon; SD. Standard deviation.

**Table 2:** HCC rates in ACLD and non-ACLD patients with different age intervals.

	Fibrotic Stage	Age, Years	Early HCC (0–3 y)/Late HCC (3–6 y)	Annual Incidence, %
All	ACLD	<60	-	1.12
		≥60	-	1.89
	Non-ACLD	<60	-	0.09
		≥60	-	0.43
DAA	ACLD	-	Early	3.26 *
		-	Late	1.39 *
		<60	-	2.26
		≥60	-	2.37
	Non-ACLD	-	Early	0.30 *
		-	Late	0.52 *
		<60	-	0.11
		≥60	-	0.45
IFN	ACLD	-	Early	1.15 *
		-	Late	0.87 *
		<60	-	0.84
		≥60	-	1.45
	Non-ACLD	-	Early	0.12 *
		-	Late	0.14 *
		<60	-	0.09
		≥60	-	0.40

* Average annual HCC incidence in 0–3 years or 3–6 years from end-of-treatment. ACLD, advanced chronic liver disease; DAA, direct-acting antivirals; HCC, hepatocellular carcinoma; IFN, interferon; N, number.

**Table 3:** Univariate and multivariate Cox regression model for HCC.

				Univariate		Multivariate	
	N	Event	IR per 10^2^ py	HR (95%CI)	*p* Value	HR (95%CI)	*p* Value
Age <60 years	2665	74	0.34	Referent			
≥60	2141	134	1.25	3.382 (2.523–4.532)	<0.001	1.888 (1.263–2.778)	0.001
Female	2476	82	0.54	Referent			
Male	2330	126	0.73	1.472 (1.113–1.945)	0.007	2.481 (1.770–3.476)	<0.001
BMI, kg/m^2^	3917	171		1.058 (1.019–1.099)	0.003	1.056 (1.012–1.102)	0.013
HCV RNA, log_10_IU/ml	4456	182		1.021 (0.881–1.183)	0.784		
Genotype 1	2610	131	0.78	Referent			
2	1893	65	0.48	0.638 (0.474–0.859)	0.003	0.799 (0.559–1.143)	0.220
3	40	1	0.46	0.569 (0.080–4.067)	0.574	0.718 (0.099–5.189)	0.743
others	140	4	0.69	0.782 (0.289–2.118)	0.629	0.503 (0.123–2.053)	0.339
Antiviral agents							
IFN-based	1981	102	0.44	Referent			
DAA	2825	106	1.14	2.264 (1.607–3.191)	<0.001	1.434 (0.875–2.351)	0.152
ALT, U/L	4806	208		1.000 (0.998–1.001)	0.779		
Total bilirubin, mg/dL	4526	197		1.266 (1.167–1.373)	<0.001	1.054 (0.860–1.290)	0.613
Albumin, g/dL	4311	187		0.291 (0.236–0.358)	<0.001	0.631 (0.429–0.927)	0.019
Platelet, 10^3^/uL	4806	208		0.986 (0.983–0.989)	<0.001		
AFP <6, ng/mL	3019	52	0.29	Referent			
≥6	1736	155	1.13	4.471 (3.262–6.128)	<0.001	3.034 (2.005–4.591)	<0.001
Diabetic mellitus, no	4115	171	0.59	Referent			
yes	691	37	1.01	1.547 (1.083–2.210)	0.017	0.996 (0.653–1.531)	0.987
Fatty liver, no	2488	129	0.75	Referent			
yes	2290	79	0.52	0.657 (0.497–0.870)	0.003	0.767 (0.539–1.091)	0.140
FIB-4	4806	208		1.142 (1.123–1.161)	<0.001	1.056 (1.020–1.093)	0.002
Non-ACLD	3068	35	0.17	Referent			
ACLD	1738	173	1.53	9.379 (6.518–13.495)	<0.001	3.297 (2.012–5.403)	<0.001

ACLD, advanced chronic liver disease; AFP, alpha-fetoprotein; ALT, alanine aminotransferase; BMI, body mass index; DAA, direct-acting antivirals; HbA1c, glycohemoglobin; HCV, hepatitis C virus; HR, hazard ratio; IFN, interferon; IR, incidence rate; py, person-year.

**Table 4:** Multivariate Cox regression model for HCC in IFN-and DAA-treated patients.

	IFN		DAA	
	Multivariate		Multivariate	
	HR (95%CI)	*p* Value	HR (95%CI)	*p* Value
Age ≥ 60 years	3.247 (1.354–3.730)	0.002		
Male			2.447 (1.640–3.651)	<0.001
AFP ≥ 6 ng/mL	1.962 (1.086–3.542)	0.025	3.086 (1.915–4.974)	<0.001
FIB-4			1.056 (1.019–1.095)	0.003
ACLD	3.806 (1.928–7.512)	<0.001	3.434 (1.813–6.506)	<0.001

ACLD, advanced chronic liver disease; AFP, alpha-fetoprotein; DAA, direct-acting antivirals; IFN, interferon.

## Data Availability

The data presented in this study are available on request from the corresponding author.

## Supplementary Materials

The following supporting information can be downloaded at https://www.mdpi.com/article/10.3390/v16091485/s1, Table S1: Comparisons of baseline clinical characteristics between patients treated with DAA and IFN after propensity score matching. Table S2: Univariate and multivariate Cox regression model for HCC after propensity score matching. Table S3: Univariate and multivariate Cox regression model for early HCC (<3 years). Table S4: Univariate and multivariate Cox regression model for late HCC (3-6 years). Figure S1: Flowchart of patient recruitment. Figure S2: Scheme for time scale in Cox regression analysis to investigate early HCC and late HCC predictors. Figure S3: The cumulative HCC incidences stratified by age ≥ 60 and < 60 years (A) in ACLD (B) in non-ACLD. Figure S4: The comparisons of the HCC risk within three years (early onset) and after three years (late onset). (A) DAA group (B) IFN group.
