# Supplementary material for: Hepatocellular Carcinoma Incidences and Risk Factors in Hepatitis C Patients: Interferon versus Direct-Acting Agents
Source: Viruses. 2024 Sep 18;16(9):1485. doi: 10.3390/v16091485 (PMC11440110; doi:10.3390/v16091485)
Supplement: Supplementary file 1 [file viruses-16-01485-s001.zip › Supplementary table_20240813.pdf]

Supplementary Table S1. Comparisons of baseline clinical characteristics between patients treated with DAA and IFN after propensity score matching

	**DAA** N=1317	**IFN-based** N=1317	P value
Age, years (SD)	55 (11)	55 (10)	0.606
Male, n (%)	673 (51)	686 (52)	0.612
BMI, kg/m^2 (IQR)	25 (22-27)	24 (22-26)	0.066
HCV RNA, log_{10}IU/ml (IQR)	6.0 (5.3-6.6)	6.0 (5.2-6.6)	0.378
Genotype 1, n (%)	664 (50)	673 (51)	0.726
ALT, U/L (IQR)	56 (31-77)	88 (53-145)	<0.001
Total bilirubin, mg/dL (IQR)	0.7 (0.5-0.9)	0.8 (0.6-1.0)	<0.001
Albumin, g/dL (IQR)	4.4 (4.1-4.5)	4.5 (4.3-4.7)	<0.001
Platelet, 10^3/uL (IQR)	185 (137-238)	170 (135-206)	<0.001
AFP, ng/mL (IQR)	4 (3-7)	5 (4-9)	<0.001
HbA1c (IQR)	5.7 (5.4-6.2)	5.6 (5.3-6.1)	0.007
Diabetic mellitus, n (%)	189 (14)	143 (11)	0.007
Fatty liver, n (%)	653 (50)	677 (52)	0.413
FIB-4 (IQR)	1.95 (1.20-3.53)	2.24 (1.46-3.37)	0.062
ACLD, n (%)	485 (37)	455 (35)	0.222
Follow-up, months (IQR)	42 (28-49)	145 (105-172)	<0.001

Numerical variables were expressed as mean ± standard deviation or median (interquartile range) according to their normality. ACLD, advanced chronic liver disease (defined as FIB-4 ≥3.25 and/or ultrasound signs of cirrhosis); AFP, alpha-fetoprotein; ALT, alanine aminotransferase; BMI, body mass index; DAA, direct acting antivirals; HbA1c, glycohemoglobin; HCV, hepatitis C virus; IFN, interferon; SD. Standard deviation

Supplementary Table S2: Univariate and multivariate cox regression model for HCC after propensity score matching

	Univariate		Multivariate	
	HR (95%CI)	P value	HR (95%CI)	P value
Age <60 years	Referent			
≥60	2.814 (1.924-4.117)	<0.001	1.678 (1.029-2.737)	0.038
Female	Referent			
Male	1.747 (1.177-2.594)	0.006	2.464 (1.503-4.041)	<0.001
BMI, kg/m^2	1.070 (1.015-1.128)	0.012	1.060 (0.997-1.126)	0.062
HCV RNA, log$_{10}$IU/ml	1.054 (0.873-1.272)	0.586		
Genotype 1	Referent			
2	0.483 (0.318-0.734)	<0.001	0.489 (0.283-0.846)	0.011
3	-	-	-	
others	0.549 (0.134-2.240)	0.403	0.405 (0.056-2.943)	0.371
Antiviral agents				
IFN-based	Referent			
DAA	1.775 (1.127-2.797)	0.013	1.293 (0.710-2.355)	0.401
ALT, U/L	1.000 (0.998-1.002)	0.721		
Total bilirubin, mg/dL	1.551 (1.243-1.935)	<0.001	0.970 (0.662-1.421)	0.876
Albumin, g/dL	0.243 (0.166-0.357)	<0.001	0.483 (0.289-0.808)	0.006
Platelet, 10^3/uL	0.988 (0.984-0.992)	<0.001		
AFP <6, ng/mL	Referent			
≥6	3.992 (2.591-6.150)	<0.001	2.554 (1.427-4.572)	0.002
Diabetic mellitus, no	Referent			
yes	1.599 (0.974-2.624)	0.064		
Fatty liver, no	Referent			
yes	0.729 (0.498-1.037)	0.104		
FIB-4	1.227 (1.172-1.284)	<0.001		
Non-ACLD	Referent			
ACLD	8.882 (5.411-14.581)	<0.001	3.917 (1.966-7.807)	<0.001

ACLD, advanced chronic liver disease; AFP, alpha-fetoprotein; ALT, alanine aminotransferase; BMI, body mass index; DAA, direct acting antivirals; HbA1c, glycohemoglobin; HCV, hepatitis C virus; HR, hazard ratio; IFN, interferon

Supplementary Table S3: Univariate and multivariate cox regression model for early HCC (<3 years)

	N	Event	Univariate		Multivariate	
			HR (95%CI)	P value	HR (95%CI)	P value
Age <60 years	2665	32	Referent			
≥60	2141	83	3.348 (2.227-5.034)	<0.001	1.688 (1.034-2.756)	0.036
Female	2476	44	Referent			
Male	2330	71	1.724 (1.183-2.510)	0.005	2.785 (1.824-4.253)	<0.001
BMI, kg/m^2	3917	103	1.064 (1.016-1.113)	0.008	1.065 (1.012-1.120)	0.015
HCV RNA, log_{10}IU/ml	4456	113	1.034 (0.854-1.252)	0.731		
Genotype 1	2610	76	Referent			
2	1893	33	0.910 (0.857-0.967)	0.002	0.741 (0.466-1.179)	0.206
3	40	1	1.325 (0.966-1.818)	0.081	1.041 (0.143-7.596)	0.968
others	140	2	1.856 (1.856-2.205)	<0.001	0.294 (0.040-2.137)	0.227
Antiviral agents						
IFN-based	1981	25	Referent			
DAA	2825	90	2.904 (1.864-4.525)	<0.001	2.373 (1.147-4.911)	0.020
ALT, U/L	4806	115	1.000 (0.998-1.002)	0.972		
Total bilirubin, mg/dL	4526	112	1.261 (1.153-1.378)	<0.001	1.076 (0.872-1.327)	0.496
Albumin, g/dL	4311	107	0.250 (0.190-0.330)	<0.001	0.765 (0.476-1.231)	0.271
Platelet, 10^3/uL	4806	115	0.983 (0.980-0.987)	<0.001		
AFP <6, ng/mL	3019	52	Referent			
≥6	1736	83	4.533 (3.015-6.815)	<0.001	2.610 (1.603-4.251)	<0.001
Diabetic mellitus, no	4115	88	Referent			
yes	691	27	1.868 (1.214-2.875)	0.005	1.212 (0.747-1.966)	0.437
Fatty liver, no	2488	79	Referent			
yes	2290	36	0.488 (0.329-0.724)	<0.001	0.647 (0.411-1.091)	0.060
FIB-4	4806	115	1.142 (1.121-1.164)	<0.001	1.070 (1.031-1.110)	<0.001
Non-ACLD	3068	16	Referent			
ACLD	1738	173	11.205 (6.608-19.000)	<0.001	4.239 (2.155-8.337)	<0.001

ACLD, advanced chronic liver disease; AFP, alpha-fetoprotein; ALT, alanine aminotransferase; BMI, body mass index; DAA, direct acting antivirals; HbA1c, glycohemoglobin; HCV, hepatitis C virus; HR, hazard ratio; IFN, interferon; IR, incidence rate; py, person-year

Supplementary Table S4: Univariate and multivariate cox regression model for late HCC (3-6 years)

			Univariate		Multivariate	
	N	Event	HR (95%CI)	P value	HR (95%CI)	P value
Age <60 years	2084	18	Referent			
≥60	1454	33	3.669 (2.057-6.545)	<0.001	1.979 (1.022-3.830)	0.043
Female	1819	23	Referent			
Male	1719	28	1.126 (0.648-1.956)	0.674		
BMI, kg/m^2	2745	37	1.065 (0.981-1.157)	0.134		
HCV RNA, $\log_{10}$IU/ml	3199	43	1.108 (0.812-1.512)	0.518		
Genotype 1	1929	31	Referent			
2	1409	16	0.891 (0.831-0.955)	0.001	0.784 (0.413-1.487)	0.456
3	23	0	0.891 (0.590-1.344)	0.581	-	-
others	81	2	1.475 (1.178-1.848)	<0.001	1.387 (0.187-10.299)	0.749
Antiviral agents						
IFN-based	1862	35	Referent			
DAA	1676	16	1.374 (0.724-2.606)	0.331		
ALT, U/L	3538	51	0.999 (0.997-1.002)	0.727		
Total bilirubin, mg/dL	3273	50	1.269 (0.920-1.750)	0.147		
Albumin, g/dL	3076	46	0.309 (0.195-0.492)	<0.001	0.470 (0.220-1.004)	0.051
Platelet, 10^3/uL	3538	51	0.989 (0.983-0.994)	<0.001		
AFP <6, ng/mL	2164	12	Referent			
≥6	1327	39	4.223 (2.208-8.075)	<0.001	2.485 (1.196-5.167)	0.015
Diabetic mellitus, no	3083	44	Referent			
yes	455	7	1.218 (0.548-2.706)	0.628		
Fatty liver, no	1822	26	Referent			
yes	1710	25	0.992 (0.573-1.717)	0.976		
FIB-4	3538	51	1.122 (1.073-1.174)	<0.001	0.915 (0.802-1.043)	0.185
Non-ACLD	2270	8	Referent			
ACLD	1268	43	9.255 (4.349-19.692)	<0.001	4.605 (1.896-11.188)	<0.001

ACLD, advanced chronic liver disease; AFP, alpha-fetoprotein; ALT, alanine aminotransferase; BMI, body mass index; DAA, direct acting antivirals; HbA1c, glycohemoglobin; HCV, hepatitis C virus; HR, hazard ratio; IFN, interferon; IR, incidence rate; py, person-year
