# Supplementary figures and images for: Hepatocellular Carcinoma Incidences and Risk Factors in Hepatitis C Patients: Interferon versus Direct-Acting Agents
Source: Viruses. 2024 Sep 18;16(9):1485. doi: 10.3390/v16091485 (PMC11440110; doi:10.3390/v16091485)

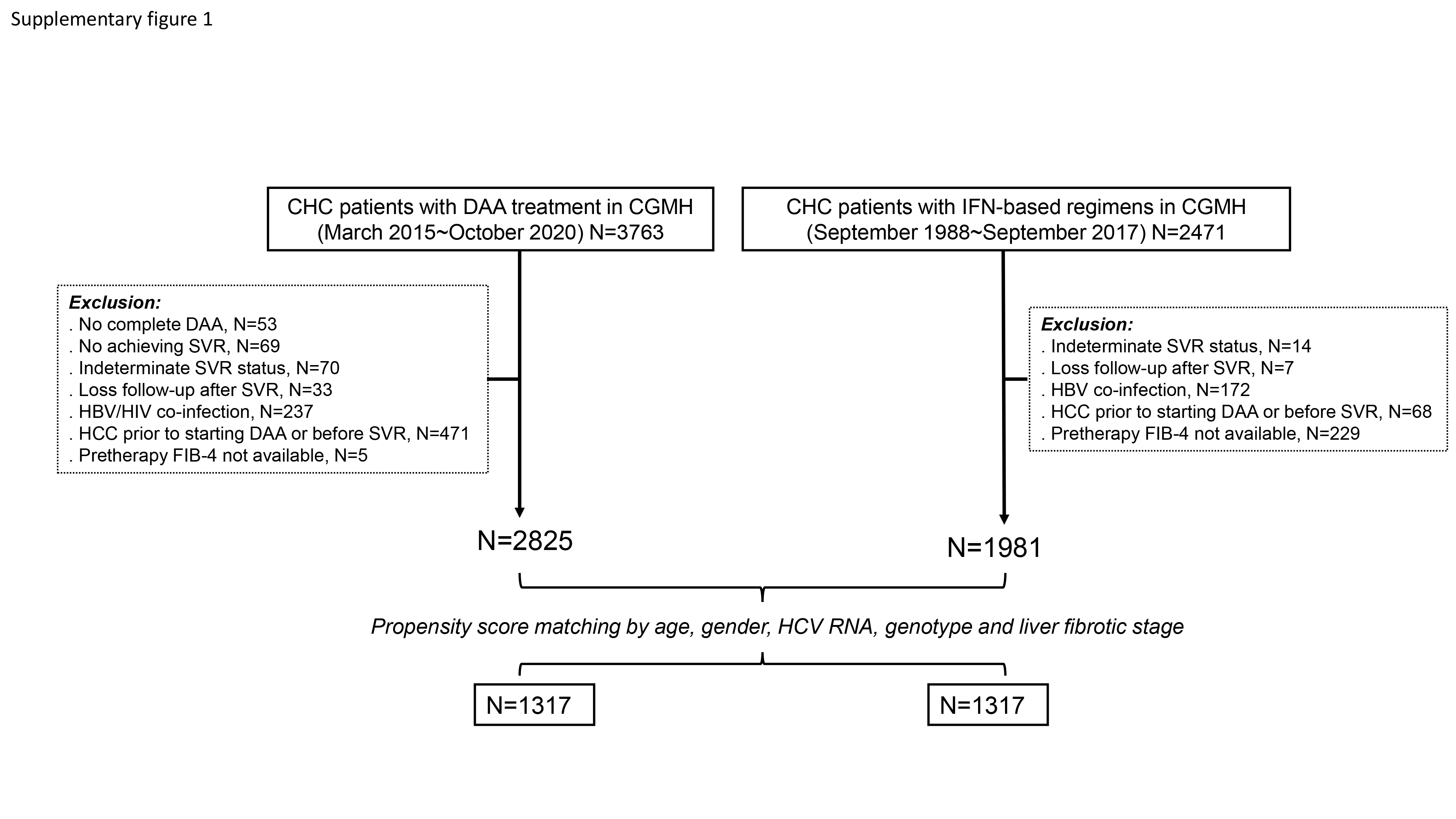

Supplement: Supplementary file 1 [file viruses-16-01485-s001.zip › supplementary figure 1.tif]

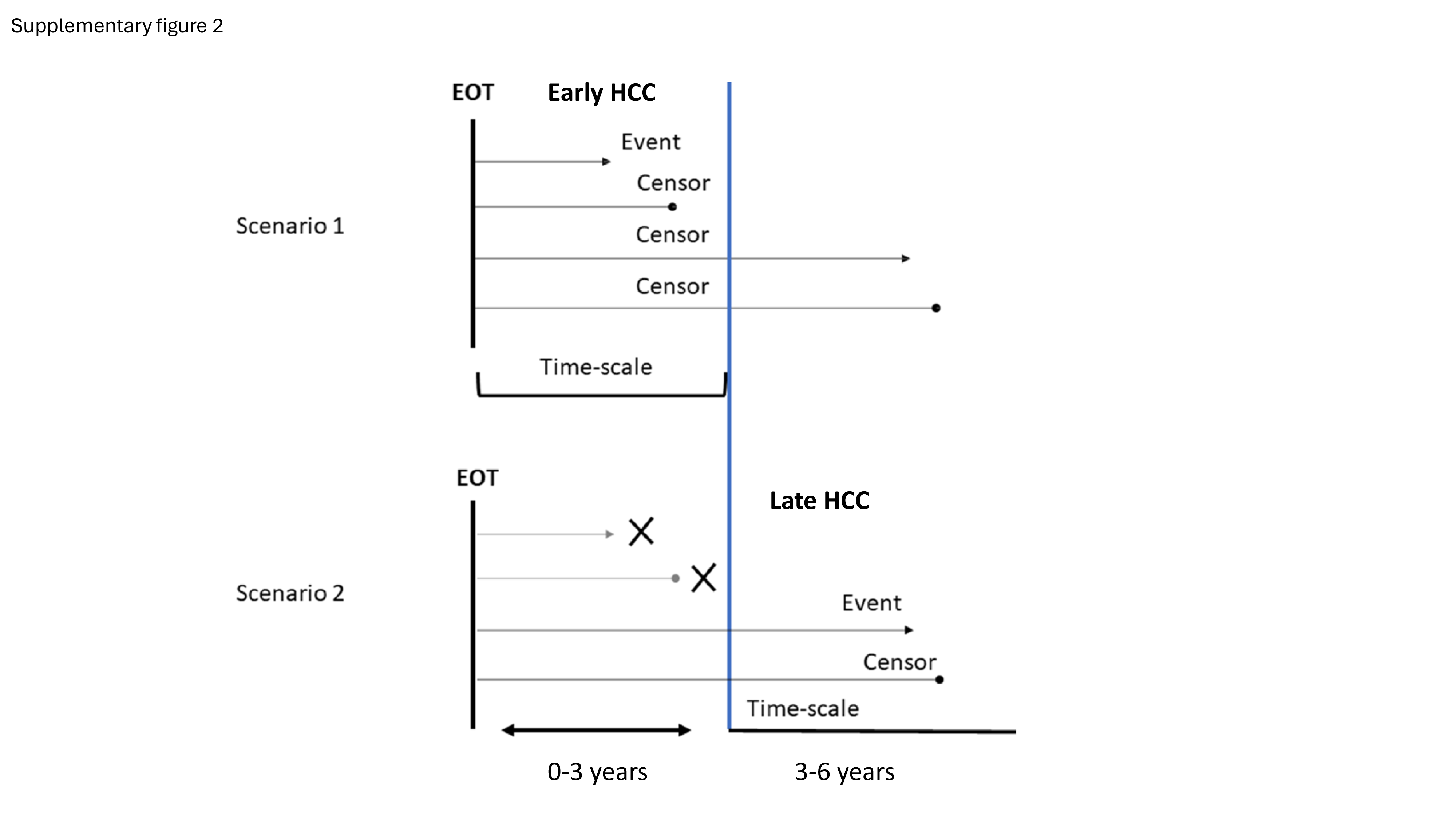

Supplement: Supplementary file 1 [file viruses-16-01485-s001.zip › Supplementary figure 2.tif]

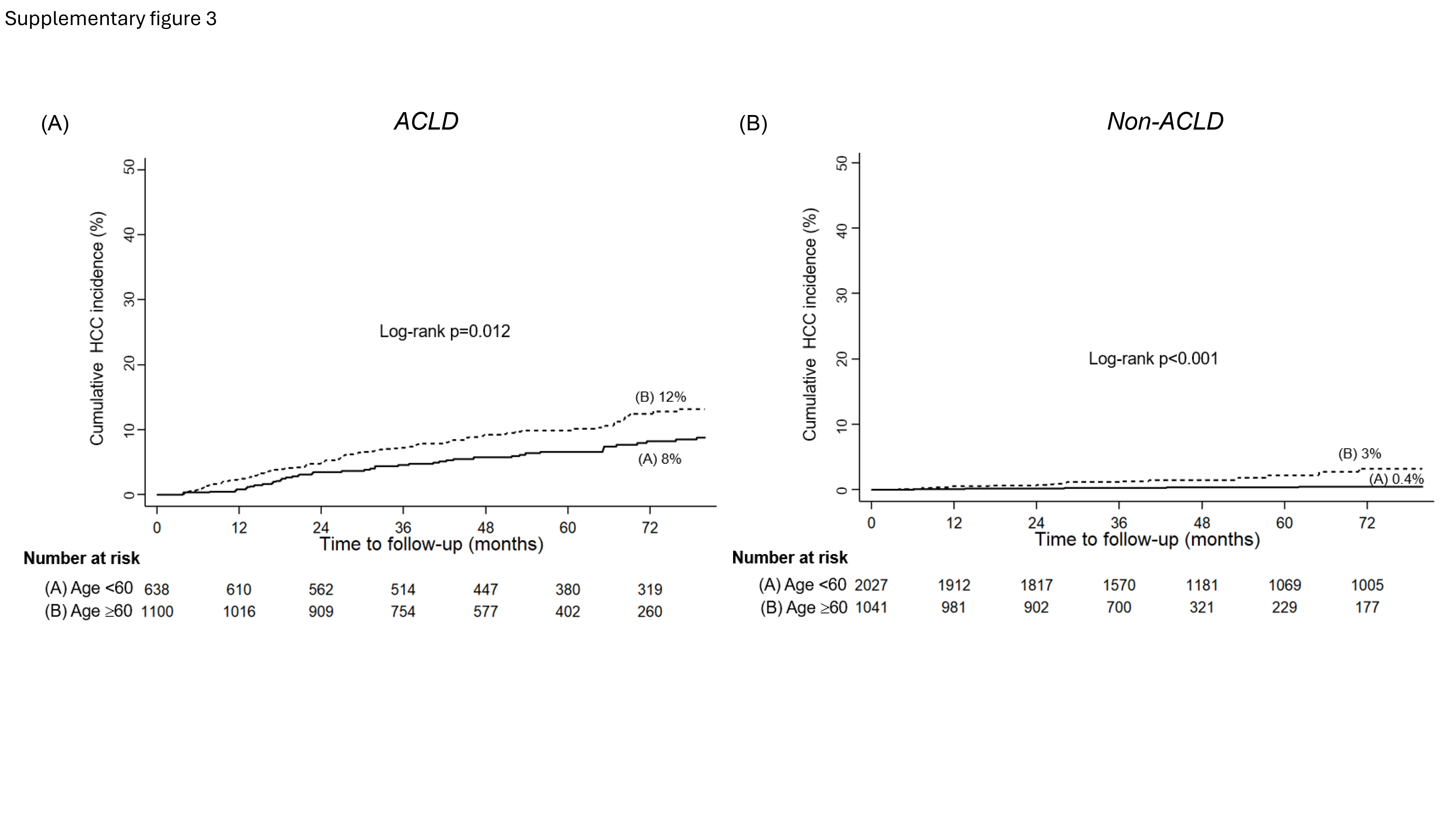

Supplement: Supplementary file 1 [file viruses-16-01485-s001.zip › Supplementary figure 3.tif]

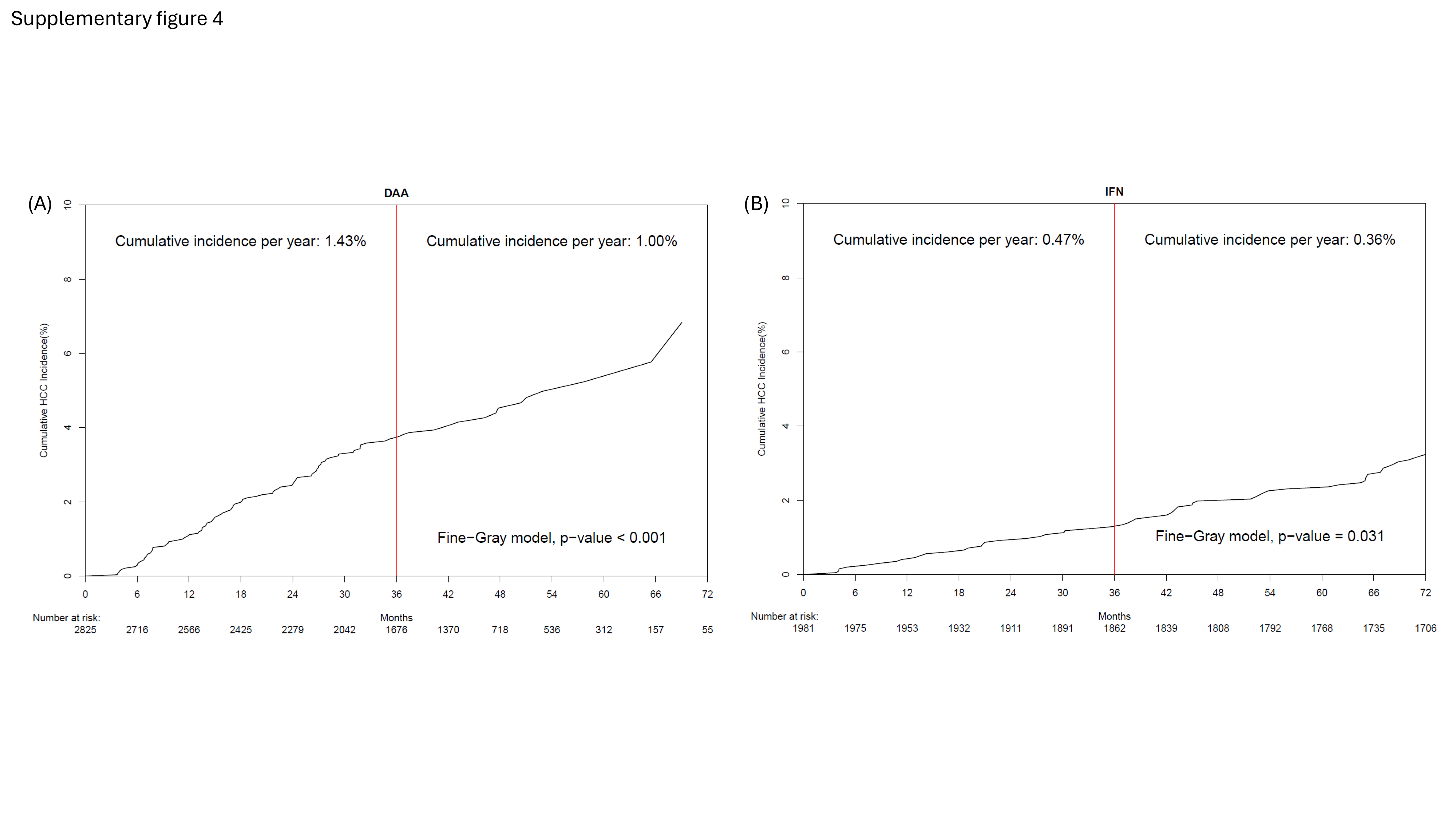

Supplement: Supplementary file 1 [file viruses-16-01485-s001.zip › Supplementary figure 4.tif]
